# SMRT sequencing of full-length transcriptome of seagrasses *Zostera japonica*

**DOI:** 10.1038/s41598-019-51176-y

**Published:** 2019-10-10

**Authors:** Siting Chen, Guanglong Qiu, Mingliu Yang

**Affiliations:** Guangxi Key Lab of Mangrove Conservation and Utilization, Guangxi Mangrove Research Center, Guangxi Academy of Sciences, Beihai, Guangxi 536007 China

**Keywords:** Bioinformatics, High-throughput screening

## Abstract

Seagrass meadows are among the four most productive marine ecosystems in the world. *Zostera japonica* (*Z*. *japonica*) is the most widely distributed species of seagrass in China. However, there is no reference genome or transcriptome available for *Z*. *japonica*, impeding progress in functional genomic and molecular ecology studies in this species. Temperature is the main factor that controls the distribution and growth of seagrass around the world, yet how seagrass responds to heat stress remains poorly understood due to the lack of genomic and transcriptomic data. In this study, we applied a combination of second- and third-generation sequencing technologies to sequence full-length transcriptomes of *Z*. *japonica*. In total, we obtained 58,134 uniform transcripts, which included 46,070 high-quality full-length transcript sequences. We identified 15,411 simple sequence repeats, 258 long non-coding RNAs and 28,038 open reading frames. Exposure to heat elicited a complex transcriptional response in genes involved in posttranslational modification, protein turnover and chaperones. Overall, our study provides the first large-scale full-length trascriptome in *Zostera japonica*, allowing for structural, functional and comparative genomics studies in this important seagrass species. Although previous studies have focused specifically on heat shock proteins, we found that examination of other heat stress related genes is important for studying response to heat stress in seagrass. This study provides a genetic resource for the discovery of genes related to heat stress tolerance in this species. Our transcriptome can be further utilized in future studies to understand the molecular adaptation to heat stress in *Zostera japonica*.

## Introduction

Currently, most transcriptomes are generated using second-generation sequencing (SGS) technologies on the Illumina platform^1^. However, SGS technologies do not yield long transcripts and information such as variable splicing cannot be obtained with SGS, restricting this technique’s utilization for generating transcriptomes^2^. Long and even full-length transcriptomes can be obtained by third-generation sequencing (TGS) platforms, such as the single-molecule real-time (SMRT) sequencer from PacBio RS (Pacific Biosciences of California, USA)^3^. There are minimal genomic resources for non-model organisms, making full-length transcriptomes particularly important for basic and applied research on gene function, regulation of gene expression and studying the evolutionary relationship among different species. The relatively high error rate of TGS may be a problem for sequence alignments and bioinformatics analyses, but could be corrected by highly accurate SGS reads^2^. Therefore, a sequencing approach that combines SGS and TGS technologies provides high-quality assemblies for transcriptome studies.

Since SMRT technology can generate long length reads, it can directly sequence full length transcripts, making it advantageous for genome and *de novo* transcriptome sequencing for the following reasons. First, full-length transcripts can be obtained directly through TGS, and the structural variants that result from alternate splicing and gene fusions can be comprehensively analyzed at the transcriptional level^4^. Second, full-length transcripts can be obtained directly without assembling the transcriptome, allowing for higher quality transcritome assemblies for organisms without a reference genome. Third, TGS can detect structural variation (SV) more accurately than SGS, making it suitable for transcriptome analysis of species that are polyploids, or have high repeat sequence and high heterozygosity.

Seagrass meadows are biological purification fields in coastal seas, and are important seabed habitats for foraging, breeding and growing of marine animals, as well as maintaining the environment of offshore waters and the safety of marine fishery resources. *Z*. *japonica* is a species of seagrass endemic to Asia, and is mainly distributed in Japan, Korea and China. *Z*. *japonica* is the most widely distributed seagrass species in China’s subtropical and temperate coastal areas. In the subtropical zone, *Z*. *japonica* often appears near mangroves or coexists with *Halophila ovalis*. In the temperate zone, *Z*. *japonica* often lives near *Zostera marina* and in the intertidal zone where the water level is shallower than areas suitable for *Zostera marina*. *Z*. *japonica* is the only seagrass species that can be found both in temperate and subtropical zones in China. *Z*. *japonica* is an intertidal seagrass, and intertidal seagrass species are more susceptible to heat stress at low tide during the summer months compared to subtidal seagrass species. Currently, there is no reference genome or transcriptome available for *Z*. *japonica*.

In this study, full-length cDNA libraries were constructed from *Z*. *japonica*, and SMRT and SGS sequencing was performed to generate full-length transcripts under heat stress and control conditions. The transcriptome provides a valuable resource for future molecular biological studies in *Z*. *japonica*.

## Results

### SMRT and Illumina sequencing and error correction

In order to determine key factors that contribute to the thermotolerance of *Z*. *japonica*, we used PacBio and Illumina platforms to sequence the transcriptome of *Z*. *japonica* leaves under heat stress and control conditions. To determine the temperature and length of exposure to the heat stress that would be most relevant for the natural conditions that *Z*. *japonica* experiences, we referred to the Pearl bay water temperature monitoring data. According to our monitoring of the water temperature in the bay, the water often reached up to 40 °C at low tide during the summer daytime, and it remained at 40 °C for about one hour (Fig. S1). Between 10 am and 12 am in May and July, the temperature of the water sometimes reached up to 40 °C (Fig. S1). Our field investigation also showed that *Z*. *japonica* tends to die during this period, likely due to the extreme high temperature conditions. Global climate change strongly affects sea level, temperature and CO_2_ in the atmosphere^5^, which can change the distribution, productivity and community composition of seagrass^6^. In particular, rising sea surface temperatures are imposing increasing heat stress on seagrass^7,8^. The change of sea surface temperatures directly affects the metabolism of seagrass and the maintenance of carbon balance^9^. Temperature is the main factor controlling the growth of seagrass by affecting the biochemical processes of the organism^10^, and high temperature in the summer inhibits seagrass growth^11^. Currently, it is unclear how much ocean warming contributes to the decline of seagrass populations. Nonetheless, the patterns suggest that the high temperature conditions during the summer months are a major cause of the decline in the seagrass meadow in Pearl Bay.

Therefore, we selected the temperature and length of exposure to 40 °C for one hour to represent heat stress experienced by *Z*. *japonica*. We collected RNA samples of control and heat stress groups and generated three biological replicates for each treatment. We extracted RNA and then equally pooled the different replicates for PacBio library preparation and sequencing. The three biological replicates of each treatment were used for Illumina sequencing and the mixed RNA was used for SMRT sequencing.

**Figure 1:**
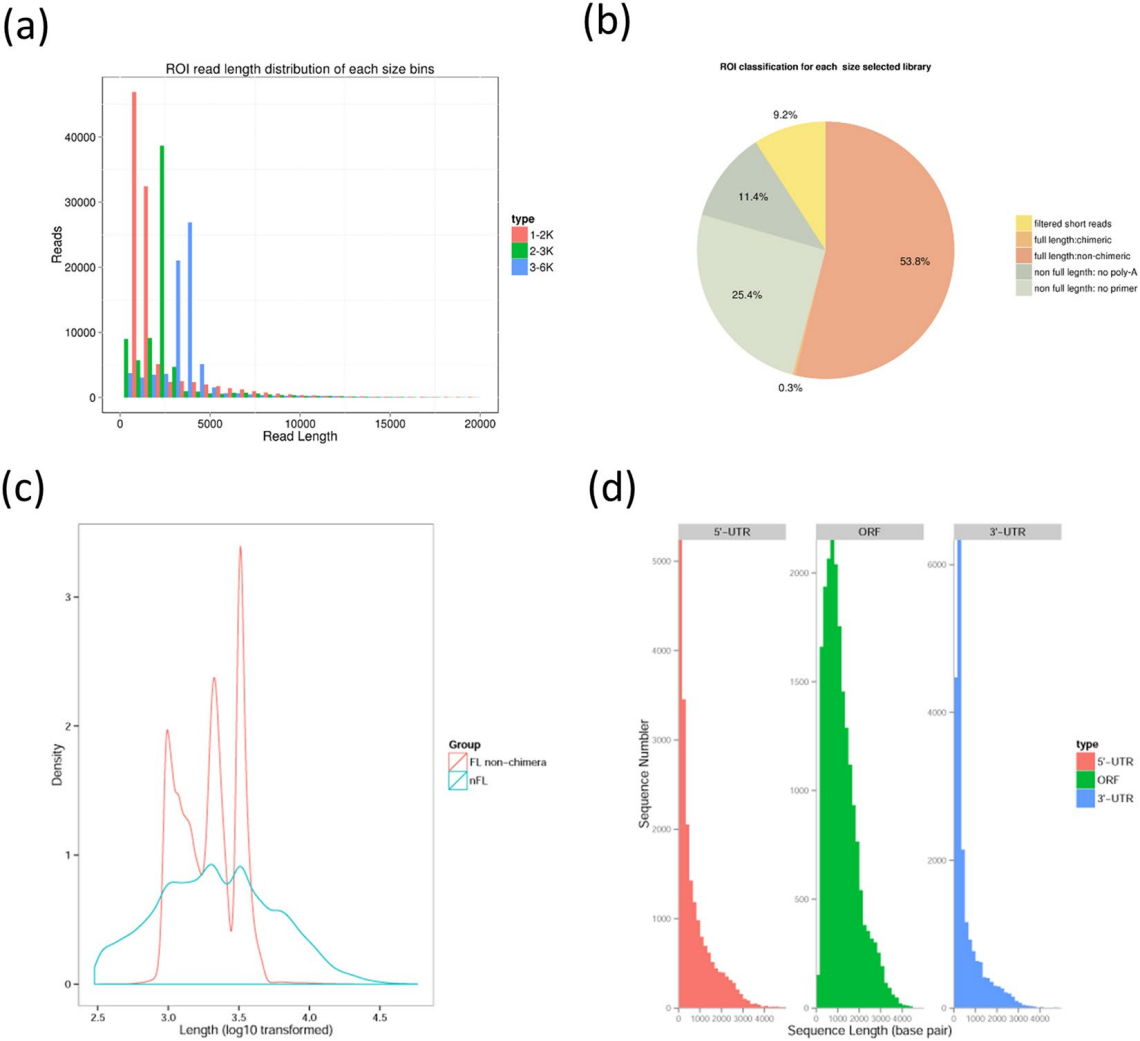
Summary of PacBio RS II SMRT sequencing. (**a**) ROI length distribution in *Z*. *japonica* from different PacBio libraries with fractionated sizes of 1–2, 2–3, and 3–6 kb. **(b)** Proportion of different types of PacBio reads in *Z*. *japonica*. (**c**) Categorization of ROIs after error correction using Illumina short reads according to the locations of the erroneous fragments. **(d)** Number and length distributions of the 20,032 non-redundant full-length transcripts.

To obtain a representative full-length transcriptome for *Z*. *japonica*, we sequenced total RNA samples from the two temperature treatments mixed together with SMRT sequencing using the Pacific RSII sequencing platform with the latest P6-C4 chemistry and four SMRT cells. We obtained 601,168 polymerase reads, yielding 276,315 reads of inserts (ROI) with mean length of 2,528 bp, quality of 0.93, and 11.00 passes on the basis of full passes of ≥0 and quality of >0.75. Of the total reads, 148,644 (54%) were full-length reads containing the 5′ primer, 3′ primer and the poly (A) tail (Data S1). In addition, 148,644 full length readsnon-chimeric (FLNC) sequences were identified (Table 1). To identify transcripts that were as long as possible, high-quality RNA was used for Iso-Seq (see Methods for details). To avoid loading bias, where shorter transcripts are preferential sequenced, three size-fractionated, full-length cDNA libraries (1–2 kb, 2–3 kb, and 3–6 kb) were constructed and sequenced in four SMRT cells (2, 1, and 1 cell, respectively; Fig. 1a). We obtained 276,315 reads of insert (total bases: 698,635,440), including 53.8% of full-length non-chimeric and 36.8% of non-full-length reads (Fig. 1b–d and Table 1).

**Table 1:** Summary of PacBio- and Illumina RNA sequencing reads.

Reads of insert of PacBio sequencing	276,315
Read Bases of Insert of PacBio sequencing (bp)	698,635,440
Reads of Illumina sequencing for correction	137,457,662
Bases of Illumina sequencing for correction (bp)	41,040,266,530
Number of non-full-length PacBio reads	101,445
Number of FLNC PacBio reads	148,644
Average length of FLNC PacBio reads (bp)	2,181
Number of non-redundant transcripts after correction	29,058
N50 of non-redundant full-length transcripts after correction (bp)	3,137
Mean of non-redundant transcripts after correction (bp)	2,469
Number of non-redundant full-length transcripts after correction	20,032

We used an isoform-level clustering algorithm, Iterative Clustering for Error Correction (ICE)^12^, to improve consensus accuracy and polished full-length consensus sequences from ICE using RS_IsoSeq (v2.3.0). We obtained 58,134 consensus isoforms, of which 46,070 were high-quality consensus transcript sequences. Short-read Illumina sequencing of control and heat stress samples was conducted to quantify the Iso-Seq non-redundant isoforms. The nonredundant transcript isoforms were used in subsequent analyses (Fig. S2).

SMRT sequencing has a high error rate, and it is necessary to correct the errors by iterative clustering of circular-consensus reads and correction with high-quality SGS short reads. To identify genes that regulate thermotolerance in *Z*. *japonica*, we sequenced the transcriptomes of *Z*. *japonica* leaves from individuals that were exposed to control and high temperature conditions. To this end, three experimental units were maintained at the control temperature of 25 °C, and the heat treated samples were exposed to as 40 °C for 1 hour. cDNA libraries were prepared from both treatment groups, and deep RNA sequencing was conducted using an Illumina HiSeq X platform. In total, 22,006,928 and 23,812,293 clean reads (total bases: 6,571,940,131 and 7,108,148,713, respectively) were obtained and used to correct the sequencing errors of SMRT in the control group and the heat stress group, respectively (Table S1, Table 1). Redundant transcripts were removed after errors were corrected. In total, we obtained 29,058 non-redundant transcripts for *Z*. *japonica* (N50: 3,137 bp; mean: 2,469 bp; Fig. S3).

### Transcript clustering analysis

A total of 59,675 consensus isoforms were obtained, including 46,070 high-quality isoforms and 12,064 low-quality isoforms. The ICE clustering results are shown in Table 2. After removing redundant sequences from the high-quality transcripts, 29,058 transcripts were obtained.

**Table 2:** ICE clustering analysis.

Samples	Size	Number of consensus isoforms	Average consensus isoforms read length	Number of polished high-quality isoforms	Number of polished low-quality isoforms	Percent of polished high-quality isoforms (%)
T01	0–1 kb	3,083	915	2,849	227	92.41%
T01	1–2 kb	22,705	1,455	19,704	2,734	86.78%
T01	2–3 kb	14,780	2,335	10,501	3,102	71.05%
T01	3–6 kb	18,571	3,513	12,995	5,542	69.97%
T01	>6 kb	536	9,004	21	459	3.92%
T01	All	59,675	2,353	46,070	12,064	77.20%

Size: insert fragment size of cDNA libraries; Number of consensus isoforms: the number of consensus isoforms obtained from ICE clustering analysis. Average consensus isoforms read length: sequence length of the consensus isoform; Number of polished high-quality isoforms: the number of high-quality transcripts; Number of polished low-quality isoforms: the number of low-quality transcripts; Percent of polished high-quality isoforms (%): percentage of high-quality transcripts in consensus isoform.

### Alternatively spliced isoforms

There are a variety of ways pre-mRNA can be alternatively spliced, where the same mRNA can be translated into different proteins with potentially divergent biological functions. In our dataset, alternatively spliced isoforms were obtained through the IsoSeq_AS_de_novo script^13^. This method compares sequences using BLAST, and if the comparison satisfies the following conditions, it is considered a candidate alternative splicing event. First, the two sequences are larger than 1000 bp, and there are two High-scoring Segment Pairs in the comparison. Second, the alternative splicing gap is greater than 100 bp, and the distance from the 3′/5′end is at least 100 bp. We could not identify the type for the detected alternatively spliced transcript isoforms because of the lack of a reference genome, and 909 alternative splicing events were identified for *Z*. *japonica* (Data S2).

### Simple sequence repeats (SSRs) analysis

SSR markers have the advantages of codominance, high reproducibility, abundant polymorphisms and easy detection^14^. Here, we detected 15,411 SSRs and 10,399 SSR-containing sequences from *Z*. *japonica* using 29,047 sequences (71,734,071 bp), and found that most SSRs had mono-, di-, or tri-nucleotide repeats (Table 3; Data S3). The number of sequences containing more than one SSR was 3,151, and 1,740 SSRs were in the compound formation. With regards to repeats, there were 7,117 mono-nucleotides, 1,705 di-nucleotide, 2,806 tri-nucleotide, 381 tetra-nucleotide, 128 penta-nucleotide and 206 hexa-nucleotide repeats in the transcriptome. Considering the high assembly quality of SMRT-derived sequences, these SSRs will serve as useful tools for analyzing genetic diversity, constructing genetic maps and identifying the location of genes in *Z*. *japonica*.

**Table 3:** SSRs predicted from the *Z*. *japonica* transcriptome

Total number of sequences examined	29,047
Total size of examined sequences (bp)	71,734,071
Total number of identified SSRs	15,411
Number of SSR containing sequences	10,399
Number of sequences containing more than 1 SSR	3,151
Number of SSRs present in compound formation	1,740
Mono- nucleotide	7,117
Di- nucleotide	1,705
Tri- nucleotide	2,806
Tetra- nucleotide	381
Penta- nucleotide	128
Hexa- nucleotide	206

### Long non-coding RNA (LncRNA) prediction

**Figure 2:**
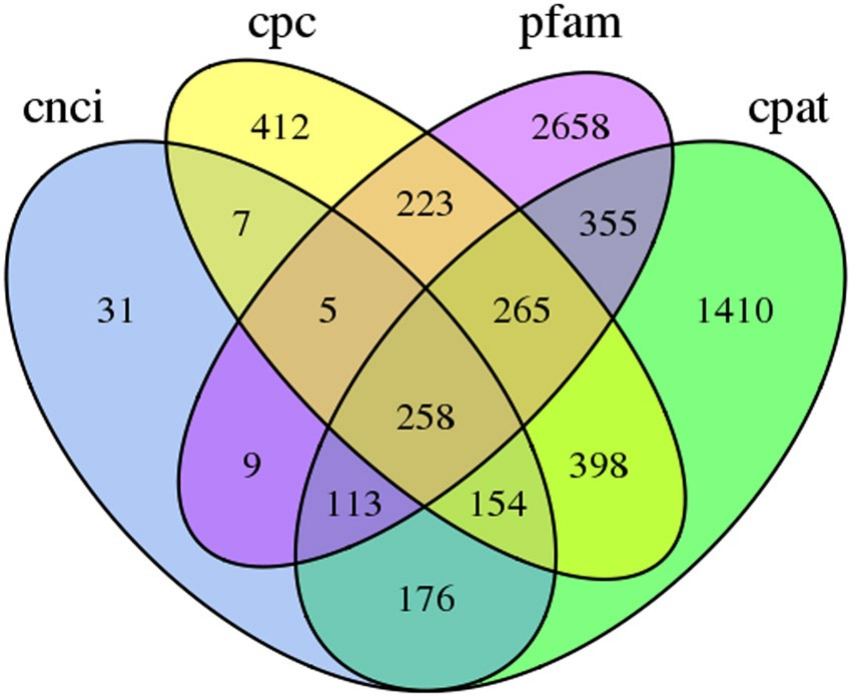
Venn diagram of the number of lncRNAs predicted by CPC, CNCI, CPAT and pfam.

LncRNAs are widely involved in physiological processes. LncRNAs regulate gene expression at the level of transcription and post transcription by epigenetic processes, and affect processes such as chromosome dose compensation, genomic imprinting, target imitation, and functional protein transport. We used CPC (Coding Potential Calculator), CNCI (Coding-Non-Coding Index), CPAT (Coding Potential Assessment Tool) and Pfam (Protein family) to identify lncRNAs from the 29,058 PacBio Iso-Seq isoforms, and identified 258 lncRNAs in *Z*. *japonica* (Data S4, Fig. 2).

The length of lncRNAs varied from 449 bp to 7,959 bp, with the majority (>71%) of lncRNAs having a length ≤3,000 bp. The mean length was 2,115 bp, which was much shorter than the mean length of all isoforms (2,469 bp).

### Coding sequence (CDS) prediction

**Figure 3:**
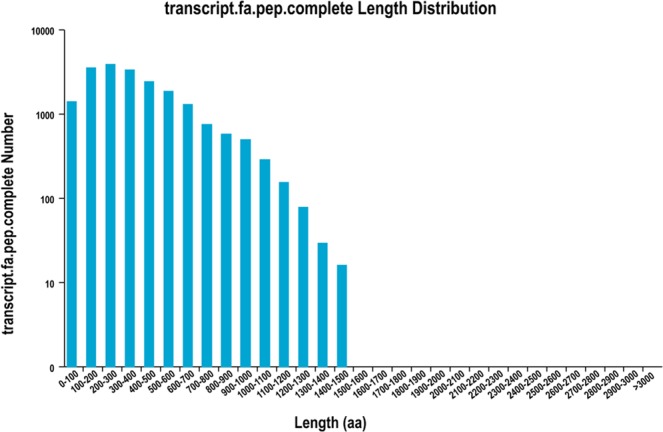
The distribution of the coding sequence lengths of the complete open reading frames. The x-axis represents the coding sequence length and the y-axis represents the number of predicted open reading frames.

In total, 28,038 ORFs were predicted using TransDecoder and their length distributions are shown in Fig. S4. Full-length transcripts were defined as transcripts that contained complete CDSs and 5′- and 3′-untranslated regions (UTRs). Of the 28,038 ORFs, 20,032 full-length transcripts were identified, and the number and length of these transcripts are listed in Fig. 1d. The distribution of the coding sequence lengths of complete ORFs is shown in Fig. 3.

### Functional annotation of transcripts

Transcripts were functionally annotated and classified using GO (Gene Ontology), KEGG (Kyoto Encyclopedia of Genes and Genomes), KOG (euKaryotic Ortholog Groups), Pfam, Swissprot, COG (Clusters of Orthologous Groups), EggNOG and NR (NCBI non-redundant protein sequences) databases using BLAST^15^ (version 2.2.26) (Table 4). In total, 12,308 transcripts were annotated in the GO database, 12,548 in KEGG, 18,986 in KOG, 23,856 in Pfam, 22,562 in Swissprot, 12,936 in COG, 27,955 in eggnog, and 28,561 in NR. Overall, 98.50% of *Z*. *japonica* transcripts were successfully annotated, and the percentage of transcripts that were annotated in a single database ranged from 42.36% to 98.29% (Table 4). These results indicate that most of the genes in our datasets are likely functional genes in *Z*. *japonica*.

**Table 4:** Annotation of transcripts using public databases.

Annotated databases	Transcript Number	Percentage
GO	12,308	42.36%
KEGG	12,548	43.18%
KOG	18,986	65.34%
Pfam	23,856	82.10%
Swiss-Prot	22,562	77.64%
COG	12,936	44.52%
eggNOG	27,955	96.20%
NR	28,561	98.29%
All annotated	28,621	98.50%
All analyzed	29,058	100.00%

GO, Gene Ontology; KEGG, Kyoto Encyclopedia of Genes and Genomes; KOG, euKaryotic Ortholog Groups; Pfam, Protein family; Swiss-Prot, a well-annotated and manually checked protein database; COG, (Clusters of Orthologous Groups; eggNOG, a database of orthologous groups and functional annotation; NR, a NCBI non-redundant protein database.

### NR annotation

**Figure 4:**
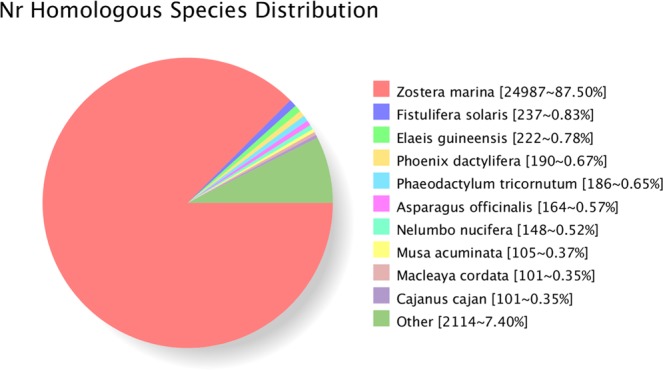
Homologous species distribution of *Zostera japonica* annotated in the NR database.

NR is a database for non-redundant proteins in NCBI, including Swissprot, Protein Information Resource, PRF (Protein Research Foundation), PDB (Protein Data Bank) protein databases and protein sequences translated from CDS sequences from GenBank and RefSeq. Species that were most closely related to *Z*. *japonica* were predicted using NR sequence alignment, and 87.50% of the *Z*. *japonica* sequences aligned to *Zostera marina* (Fig. 4).

### GO annotation and eggNOG annotation

**Figure 5:**
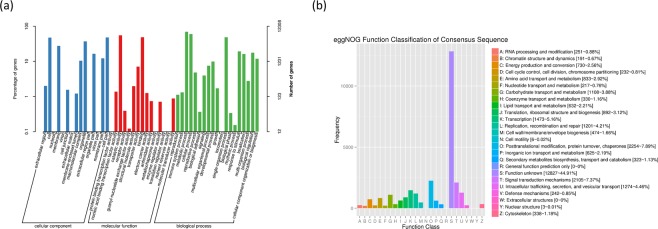
GO functional annotation and eggNOG annotation of *Zostera japonica* transcripts. (**a**) GO annotation, where blue represents cellular component, red represents molecular function, and green represents biological process. The x-axis represents GO categories, the y-axis (left) represents the percentage of genes, and the y-axis (right) represents the number of genes. **(b)** eggNOG annotations, where the x-axis represents eggNOG categories and the y-axis represents the number of transcripts.

The GO database is an international standardized classification system of gene functions, providing a set of dynamically updated standard vocabulary to describe the functional properties of genes and gene products in organisms. There are three main categories in the database, molecular function (MF), cellular component (CC) and biological process (BP), which describe the possible molecular functions of gene products, as well as the cellular environment and the biological processes they may be involved in. In this study, GO analysis revealed that the transcripts were enriched in several CC, MF and BP associated terms (Fig. 5a).

The eggNOG database is a database of orthologous groups and functional annotation. Transcripts in the *Z*. *japonica* transcriptomes were most enriched for function S, followed by function O and function T (Fig. 5b).

### Screening of differentially expressed transcripts

DESeq^16^ was used to analyze gene expression differences between control and heat treated groups to identify differentially expressed transcripts (DETs). To detect DETs, fold change (FC) ≥ 2 and false discovery rate (FDR) < 0.01 was used as the screening standard. Fold change indicates the ratio of expression between control and heat treated samples. False discovery rate was obtained by correcting the differentially significant p value. Differential expression analysis of transcriptome sequencing is an independent statistical hypothesis test for a large number of transcript expression values, therefore increasing the chance of identifying false positive DETs. To remove false positive DETs from the analysis, the Benjamini-Hochberg correction method was used to correct the significant p value obtained from the original hypothesis test, and FDR was used as the key index for screening of DETs.

To identify genes involved in the transition from normal growth temperature to heat stress, we identified 659 DETs using the RNA-seq data, which accounted for 1.10% of all isoforms identified in this study (Data S5).

### Cluster analysis, GO annotation and COG annotation of differentially expressed transcripts

**Figure 6:**
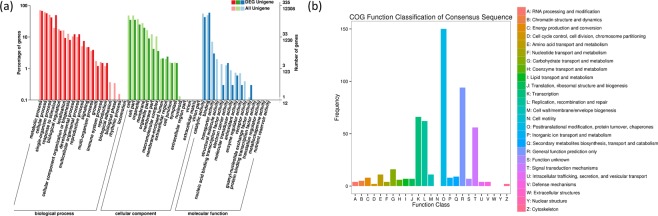
GO functional annotation and COG annotation of *Zostera japonica* DEGs. (**a**) GO annotation, where red represents biological process, green represents cellular component, and blue represents molecular function. The x-axis represents GO categories, the y-axis (left) represents the percentage of genes, and the y-axis (right) represents the number of genes. **(b)** COG annotation, where the x-axis represents COG categories and the y-axis represents the number of transcripts.

To better understand the functions of the genes expressed under different temperatures, we performed GO enrichment analysis on DETs. The DETs that were enriched under heat stress were under the GO categories of response to stimulus, reproductive process, extracellular region, membrane enclosed lumen, electron carrier activity and binding associated terms (Fig. 6a).

The COG database is a database for homologous classification of gene products. It is an early database that is often used for identifying orthologous genes by comparing protein sequences across many species. DETs were most enriched in function O (posttranslational modification, protein turnover, chaperons), followed by function R (general function prediction only) and function K (transcription) (Fig. 6b).

### Validation of differentially expressed transcripts of *Z*. *japonica* under heat stress

**Figure 7:**
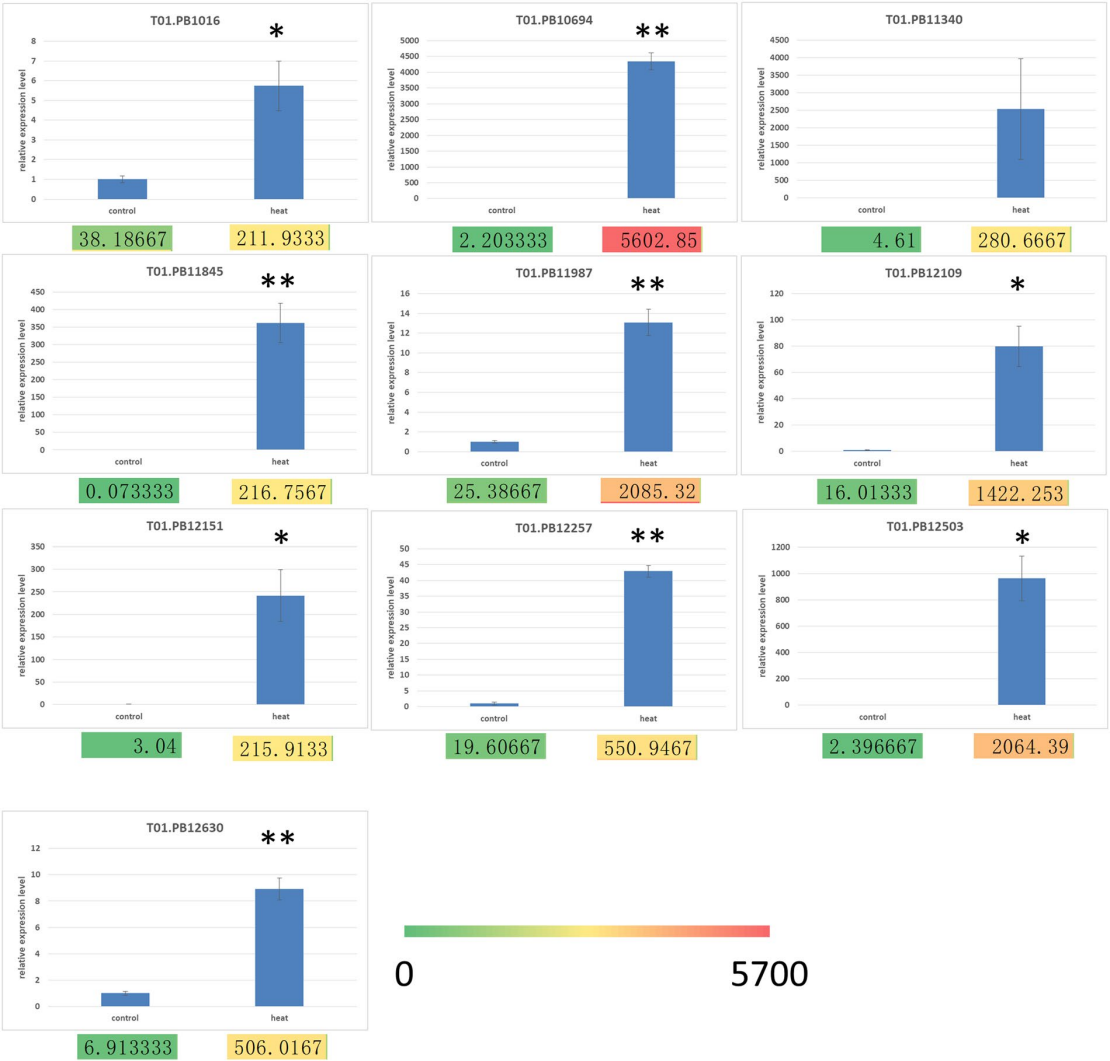
qRT-PCR validation of differentially expressed genes in *Z*. *japonica* under heat stress. Expression levels were normalized to the reference gene *actin*. Error bars indicate standard deviations of the three biological replicates. The statistical difference between means is indicated as *P < 0.05 or **P < 0.01. Colors and numbers under the bar charts refer to the FPKM of genes.

Quantitative Real-Time PCR (qRT-PCR) analysis was conducted to validate the DETs. The same total RNA sample was used for Real-Time PCR analysis. Ten genes were selected for validation (Fig. 7) and their *Arabidopsis* orthologs are listed in Table S2. All samples were normalized to the housekeeping gene *actin*. All genes showed consistent qRT-PCR expression patterns as the RNA-seq. The results showed that the results of RNA-seq technique were authentic. The expression levels of the 10 differentially expressed genes matched these of high throughput sequencing data.

### Mapping the entire RNA-seq reads from HiSeq to the *Zostera muelleri* and *Zostera marina* genome and ortholog detection

We mapped the entire RNA-seq reads from our HiSeq transcriptome to the *Zostera muelleri* and *Zostera marina* genome using Bowtie2 with parameter X 500, and the consensus mapping rates were 60% and 4%, respectively (Table S3). In the SMRT sequencing of full-length transcriptome of seagrasses *Zostera japonica*, 56076 proteins were identified, 18265 proteins had orthologs in *Zostera muelleri* (Data S6), and 16866 proteins had orthologs in *Zostera marina* (Data S7) using Blast. The standard for ortholog detection was e value < 1e-5, and the minimum coverage was >60%. The high protein mapping rate and low nucleotide mapping rate of *Zostera marina* may be attributed to more synonymous mutations in the protein coding region of *Zostera marina* and *Zostera japonica*.

## Discussion

The seagrass meadow is a typical marine ecosystem that is critical for protecting marine ecology and biodiversity. Compared to well-studied plants, little attention has been paid to seagrass despite the genomes that are available for *Zostera marina*^17^ and *Zostera muelleri*^18^. Full-length cDNA sequences are basic resources for genomic studies. In this study, full-length transcriptomes of *Z*. *japonica* was sequenced by SMRT and Illumina sequencing. However, the seagrass transcriptome is not exhaustive because of the limited biological samples. We wanted to create a general characterization of gene expression patterns, therefore we sequenced pooled RNA samples to reduce the cost of the SMRT sequencing.

SMRT sequencing can generate super-long reads under a short running time with no template amplification. It compensates for the limitations of SGS, such as the length of sequencing reads and the influence of GC content. In addition, SMRT sequencing can be used to analyze alternative splicing events. In this study, 276,315 ROI and 148,644 FLNC reads were generated after SMRT sequencing. 46,070 high-quality isoforms and 12,064 low-quality isoforms were identified through transcript clustering analysis of FLNC reads.

SMRT sequencing is characterized by its relatively high error rate^19^, and there are many computational approaches to correct the errors using short reads^19^. We sequenced the short reads for the control and heat treatment groups to correct the PacBio reads. Data from SGS were used to correct the 12,064 low-quality isoforms obtained from the PacBio reads. After removing redundant transcripts using CD-HIT, 29,058 transcripts (including 20,032 putative full-length ones) were identified in total, with 909 predicted alternative splicing events. There were 15,411 SSRs, 20,032 complete CDS regions and 258 LncRNA that were predicted in our transcriptomes, and 28,621 transcripts were annotated.

A series of annotation analyses were performed on the 29,058 high-quality transcripts. NR annotation revealed that 87.50% of the sequences were aligned to *Zostera marina*, and *Z*. *japonica* had lower similarity with palm plants (Fig. 4).

Many of the transcripts that were identified in this study were enriched in GO subcategories, such as cell, cell part, catalytic activity, binding, metabolic process and cellular process. The results of eggNOG annotation showed that the most enriched category of transcripts in the *Z*. *japonica* transcriptome was the unknown function category. The results showed that *Z*. *japonica* transcripts were related to the abovementioned functions.

LncRNAs are a group of RNA molecules that have highly conserved secondary and tertiary structures that are crucial to its biological function. Compared with animals, the function of plant lncRNA remains elusive. So far, more than 10,000 lncRNAs have been found in *Arabidopsis*, rice, wheat, maize and soybean. LncRNAs play an important role in stress response. We identified 258 lncRNAs.

Our analyses have provided a transcriptome for *Z*. *japonica*, which can facilitate molecular biological studies in *Z*. *japonica*, the most widely distributed seagrass species in China. In our transcriptomic analysis, we identified 29,058 transcripts, including alternative spliced isoforms, and we updated the *Zostera* annotation using this information. We also compared gene expression in *Z*. *japonica* leaves exposed to control and high heat temperatures, and identified many DETs under heat stress. However, the functions of these genes remain unknown. These genes are a potential resource to study thermotolerance in *Z*. *japonica* in future studies.

qRT-PCR was applied to verify the DETs. The *Arabidopsis* orthologues to these genes encode calcium binding protein, heat shock protein 70 (HSP70) and HSP90 (Table S2). DNA methylation maintains genomic integrity, and also regulates gene expression during stress response. Small RNAs can direct DNA methylation. This process involves PolIV and PolV. The promoter of AT3G50770 contains transposon insertions and is affected by DNA methylation. At higher temperatures, the methylation the promoter of AT3G50770 decreased and its expression increased^20^. HSP70’s involvement in heat shock response has been reported in *Arabidopsis*^21^, rice^22^, tobacco^23^, and pepper^21^. At5G56010 is a member of the HSP90 family that is expressed at a particularly high level in the root apical meristem, pollen and tapetum. Expression of At5G56010 is not heat-induced^24^, but induced by IAA and NaCl. Overexpression of At5G56010 reduced tolerance to heat and conferred higher tolerance to calcium in Arabidopsis. The mRNA of At5G56010 can move across different cells. The expression of T01.PB12109 was induced by high temperature, but its orthologous gene expression in *Arabidopsis* was not induced by high temperature, which may be caused by species differences.

Transcriptomics provide valuable insight into the biology of organisms, especially in non-model systems. Two previous studies have investigated the transcriptomes of seagrass under heat stress^25,26^. Franssen *et al*.^26^ studied the transcriptomic adaptation to global warming in the seagrass *Zostera marina*. Another study compared the transcriptomic responses of the seagrasses *Zostera marina* and *Nanozostera noltii* under a simulated heatwave, and found that *N*. *noltii* showed higher thermal tolerance^25^. These studies focused on predicting how seagrass species will respond to increasing climatic extremes predicted under global warming^25,26^. Here, we investigated the transcriptome of *Z*. *japonica* under heat stress and found that calcium signaling pathway and heat shock protein signaling pathway may be involved in the formation of heat tolerance in *Z*. *japonica*. Under heat stress, a specific calcium channel located on the plasma membrane of *Physcomitrella patens* opens, leading to calcium influx and activating the expression of heat shock response genes^27^. Treating plants with calcium channel blockers or chelating agents have allowed for the identification of calcium channels as one of the major heat stress receptors. Heat stress increases membrane fluidity^28^, and increased membrane fluidity with chemical reagents also initiated heat shock response^27^. Therefore, increased membrane fluidity may activate calcium channels, thereby triggering the downstream heat shock response^27^. The *Arabidopsis thaliana* genome encodes more than 40 predicted calcium channels^29^, and many calcium channels have a predicted calmodulin binding domain. For example, Arabidopsis calmodulin AtCaM3 is an essential gene for heat stress signal transduction^30^ that activates calcium/calmodulin-binding protein kinase (CBK). Calcium influx into the cell activates multiple calcium-dependent protein kinases (CDPKs) and phosphorylates MBF1c (multiprotein bridging factor 1c), which triggers the expression of heat stress response genes^31^. HSPs are a class of evolutionarily conserved polypeptides that are expressed under environmental stress and during development^32^. Under high temperature stress, HSPs act as a molecular chaperone to prevent denatured proteins from aggregating. HSPs assist denatured proteins to fold or dissolve aggregated denatured proteins^33^. Plant HSPs can be divided into five groups. In *Arabidopsis thaliana*, heat stress alters the functional status of chloroplasts and mitochondria, and changes HSP gene expression patterns^34,35^. In prokaryotic and eukaryotic organisms, HSP90 is abundant, accounting for 1%~2% of the total protein^36^, and is fundamental to the survival of eukaryotic organisms. Under heat stress, HSP90 accounts for 4%~6% of total protein content of a cell^36^. HSP90 is expressed constitutively in eukaryotes, and its constitutive expression is important for plant heat tolerance. *HSP90* is down-regulated in the *TU8* mutant of *Arabidopsis thaliana*, leading to the mutant’s thermal sensitivity^37^. On contrast, Arabidopsis seedlings show increased heat tolerance when they are treated with fungal HPS90 inhibitors, which up-regulate the expression of *HSP101* and *HSP70*^38^. Under heat stress, the activity of HSP90 decreases, and protein maintenance system is activated. HSP90 is inactivated immediately under heat stress. After heat stress, cytoplasmic HSP90 resumes its function and inhibits heat shock transcription factors, initiating a negative feedback against the expression of heat-induced genes^36^. The *Hsp70* gene family can be divided into four subfamilies that are located in cytoplasm, endoplasmic reticulum, plastid or mitochondria. Cytoplasmic *HSP70* is involved in the establishment of plant thermotolerance^39^. *HSP70* gene encodes a highly conserved chaperone protein, which exists in bacteria, plants and animals. HSP70CP1 localizes to the chloroplast in rice (*Oryza sativa*) and is fundamental to chloroplast development under heat stress. *OsHSP70CP1* is mainly expressed in photosynthetic tissues, and OsHSP70CP1 is localized to the matrix of chloroplast and up-regulated under heat stress^22^. Yeasts that carry *Ssc1* (HSP70 protein of yeast mitochondria) mutations cannot grow at higher temperature. Three rice mitochondrial *HSP70s* can restore yeast growth under heat stress to varying degrees^40^. We found that the expression of calmodulin, *HSP90* and *HSP70* genes were up-regulated under heat stress in *Z*. *japonica*, suggesting that calcium signaling pathway and heat shock protein signaling pathway may be involved in the formation of heat tolerance in *Z*. *japonica*.

## Methods

### Plant material

*Z*. *japonica* often appears in the vicinity of mangrove in the subtropics and *Z*. *japonica* is one of the most important seagrass in the coast of Guangxi. *Z*. *japonica* individuals were collected in the winter of 2017 from a southern Asian location in the Nanhai sea and Jiaodong seagrass meadows in Pearl bay, China (21°37′14.45′′N, 108°20′50.00′′E, 2017). Entire shoots with attached roots were collected. Plant materials were replanted and exposed to different temperature treatments. Fifteen plants were planted in each tank and plants were maintained in laboratory conditions for five days before heat treatment. Shoots exposed to temperature treatments were planted into 30-L plastic crates that were filled with seawater (salinity ∼15 g/kg) with a light and dark cycle of 16 and 8 h at 25 °C. The heat stress was applied after 4 hours of illumination. Three experimental units were maintained at the control temperature of 25 °C, while three other tanks were exposed to 40 °C for one hour. Seagrasses were sampled immediately after heat stress by cutting mature and epiphyte-free leaves, and samples were rinsed three times in ultrapure water to remove epiphytes, then immediately frozen in liquid nitrogen. Three independent biological replicates were collected for each treatment.

We obtained permission from the Beilun Estuary Preserve to collect samples and samples were collected in compliance with the Convention on the Trade in Endangered Species of Wild Fauna and Flora (https://www.cites.org/). Formal species identifications were performed by Guanglong Qiu. Voucher specimens are deposited in Guangxi Mangrove Research Center.

### RNA extraction and detection

Total RNAs were extracted by the Tiangen RNA preparation kits (Tiangen Biotech, Beijing, China) following the manufacturer’s protocol. We used the following methods to test for the quality of the RNA. First, RNA degradation was estimated on agarose gels. Second, RNA purity (OD260/280), concentration and absorption peaks were determined using Nanodrop. Third, the integrity was determined using Agilent 2100. The detection indexes included: RIN value, 28S/18S, baseline for spectra and 5S peak (Table S4). RNA samples were used for constructing cDNA libraries. One microgram for each RNA sample from the two temperature treatments was pooled together at an equal ratio and used for PacBio single-molecule long-read sequencing. All six samples (two temperature treatment, three biological replicates) were used for Illumina sequencing (Fig. S5).

### Illumina cDNA library construction

1 μg RNA was used to generate libraries using the NEBNext UltraTM RNA Library Prep Kit for Illumina (NEB, USA) following the manufacturer’s protocol, and index codes were added to attribute sequences to a specific sample. Eukaryotic mRNA was enriched by magnetic beads with Oligo (dT). mRNA was fragmented randomly into ~200 bp fragments by using divalent cations under elevated temperature. The first cDNA chain was synthesized by random hexamers and M-MuLV Reverse Transcriptase (RNase H-) using mRNA as the template. The second cDNA chains were synthesized by mixing buffer, dNTPs, RNase H and DNA polymerase I, and the cDNA was purified using AMPure XP beads. The cDNA was repaired at the end. After adenylation of 3′ ends, NEBNext Adaptors were ligated. AMPure XP beads were used to choose for cDNA fragments that were 200–250 bp in length. USER Enzyme (NEB, USA) was incubated with cDNA at 37 °C for 15 min followed by 5 min at 95 °C. The cDNA library was enriched by PCR using Phusion High-Fidelity DNA polymerase, universal PCR primers and index (X) primer. Finally, PCR products were purified by AMPure XP system.

### PacBio cDNA library construction

The SMART sequencing library was prepared according to the Iso-Seq protocol as described by Pacific Biosciences (P/N100-377-100-05 and P/N100-377-100-04). Full-length cDNAs of mRNAs were synthesized with SMARTer PCR cDNA Synthesis Kit. One microgram of RNA was reverse transcribed. After PCR amplification, full length cDNA was repaired at the end. The SMRT dumbbell type adaptors were ligated to the cDNA using the SMRTBell Template Prep Kit. Full-length cDNA fragments were screened using BluePippin and cDNA libraries (1–2 kb, 2–3 kb, and 3–6 kb) were constructed according to the Iso-Seq protocol. BluePippin was used for the second screening, and the sequencing libraries were obtained.

### Quality control of library and sequencing

After the construction of the sequencing libraries, the quality of the libraries was detected. First, Qubit2.0 was used to make accurate quantitative analyses. Second, the size of the libraries was detected using Agilent 2100. For SGS, the clustering of index-coded samples was performed on a cBot Cluster Generation System. After clustering, Illumina transcriptome libraries were sequenced on an Illumina Hiseq X Ten platform, and 150 bp paired-end reads were generated. For TGS, PacBio Iso-Seq libraries were prepared by annealing a sequencing primer and adding polymerase to the primer-annealed template. The template was bound to MagBeads. Sequencing was performed on a PacBio RsII instrument. Four SMRT cells were used for sequencing using P6-C4 chemistry. Both TGS and SGS was performed by Biomarker Technology Co. (Beijing, China).

### Quality filtering and error correction of PacBio long reads

The transcriptome was assembled using data from TGS, and SGS sequencing results were used to correct the TGS sequencing data and analyze gene expression levels. SMRT-Analysis software package v3.0 (https://github.com/ben-lerch/IsoSeq. 3.0/blob/master/README.md) was used for Iso-Seq data analysis. Three analytic stages were used to generate the full length transcriptome^12^. First, the ROI sequence was extracted from the original sequence of the machine using the minimum filtering requirement of 0 or greater passes of the insert and a minimum read quality of 75, and cDNA primers and poly (A) in the sequence were filtered. ROIs shorter than 50 bp were abandoned, and the sequence was divided into full-length sequence and non-full-length sequence, chimeric sequence and non-chimeric sequence according to whether there were 3′ primers, 5′ primers and Poly (A) (optional). Second, using the ICE algorithm, the full length sequences of the same isoform were clustered, and the full length sequences that were similar were also clustered, and each cluster was used to obtain a consistency sequence. Third, the Quiver algorithm was used to cluster sequences that were not full length. Using this, the consistency sequences of high quality and low quality were obtained. Full-length (FL) transcripts with post-correction accuracy above 99% were used for further analysis. Full-length non-chimeric (FLNC) reads were identified. SGS data were used to correct low quality conformance sequences using LoRDEC tool v0.6 with –k 21, -s 3, and default setting for other parameters^41^. Considering the limitation of the construction of the cDNA library^12^, the consistency sequence we screened may be incomplete because of the deletion of the 5′ terminal sequence. Therefore, we only merged the sequences that were different only in the 5′ terminal exons. The longest sequence was selected as the final transcript. Iso-Seq high quality FL transcripts were removed for redundancy using cd-hit (identity > 0.99). The resulting transcript sequences were directly used for subsequent analysis to detect lncRNA, SSR and variable splicing. SGS data were used for quantitative and differential expression analysis of transcripts.

### Quality control of SGS data

Using the sequencing by synthesis (SBS) technology, cDNA libraries were sequenced by Illumina HiSeq high throughput sequencing platform, known as the raw data, and most of the base quality score reached or exceeded Q30.

### Data statistics of SGS

Raw data (raw reads) were processed using in-house perl scripts. Clean data (clean reads) were obtained by removing reads containing adapters or polyN and low-quality reads.

### SSR analysis

MISA (MIcroSAtellite identification tool; http://pgrc.ipk-gatersleben.de/misa/) is a software for identifying SSRs. By analyzing sequences, seven types of SSRs can be identified. Only transcripts that were ≥500 bp in size were used to detect SSRs.

### Prediction of lncRNA

LncRNAs do not encode proteins, therefore transcripts were screened for protein coding potential to determine whether the transcript may be a lncRNA. CPC^42^ analysis, CNCI^43^ analysis, Pfam protein domain analysis and CPAT^44^ analysis were used to detect coding potential. Putative protein-coding RNAs were filtered out using a minimum length and exon number threshold. Transcripts that were longer than 200 nt and had more than two exons were selected as lncRNA candidates. This threshold was chosen to filter low-expression, low-confidence, and single-exon transcripts^45^. The putative lncRNAs were further filtered with CPC, CNCI, Pfam and CPAT.

### Prediction of CDSs

The TransDecoder (V3.0.0)^46^ software (https://github.com/TransDecoder/TransDecoder/releases) can identify reliable potential CDSs from transcripts based on the length of ORFs, log-likelihood score, the amino acid sequence and the Pfam database protein domain sequence using the following standards. First, a minimum length ORF is found in a transcript sequence. Second, a log-likelihood score similar to what is computed by the GeneID software is >0. Third, the above coding score is greatest when the ORF is scored in the 1st reading frame as compared to scores in the other five reading frames. Forth, if a candidate ORF is fully encapsulated by the coordinates of another candidate ORF, the longer one is reported. Fifth, optional the putative peptide has a match to a Pfam domain above the noise cutoff score.

### Functional annotation

Functional annotation of the 29,058 transcripts was performed using BLAST^15^ (version 2.2.26) to compare trascripts againt the NR, Swissprot, GO, COG, KOG, Pfam, KEGG databases with an E-value threshold of 10^−5^. The functional information was assigned to the best matched sequence.

### Quantification of gene expression levels

Gene expression levels were estimated using RSEM^47^. First, clean data were mapped back onto the assembled transcriptome. Second, readcount was obtained from the mapping results. Gene expression levels were estimated by fragments per kilobase of transcript per million fragments mapped (FPKM).

### Differential expression analysis

Differential expression analysis between heat treatment group and control group was performed using the DESeq R package (1.10.1)^48^. P values were adjusted using the Benjamini and Hochberg’s approach^48^. Genes with Fold Change ≥2 and FDR < 0.01 that were identified by DESeq were assigned as differentially expressed. K-means clustering was conducted using the Pearson correlation of gene expression profiles^49^.

### GO enrichment analysis

GO enrichment analysis of the DEGs was implemented using the GOseq R packages^50^. GO enrichment analysis was performed using topGO^51^, and the negative log 10 transformed P values were visualized using heatmaps^52^.

### qRT-PCR analysis

Ten DEGs were randomly chosen to validate the RNA-seq data. Total RNA samples were isolated from *Z*. *japonica* leaves for qRT-PCR with the RNAprep Pure Plant Kit (TIANGEN, Beijing, China) as previously described. First-strand cDNA was reverse transcribed using the PrimeScript^TM^ RT reagent Kit with gDNA Eraser (Takara, Dalian, China). qRT-PCR was performed using SYBR Premix Ex Taq^TM^ (Perfect Real Time) and ROX plus (Takara, Dalian, China). All samples were normalized to *Z*. *japonica actin* to determine the isoform expression level. All primers were designed from the PacBio-seq isoform sequences and are listed in Supplementary Table S5. The conditions for the qRT-PCR amplification were as follows: incubation at 95 °C for 30 sec, 40 cycles of 95 °C for 5 sec and 60 °C for 30 sec. The specificity of the primer amplicons was tested using a melting curve analysis and PCR products were verified by sequencing. The amplification was carried out in a thermal cycler (MJ Mini^TM^ Personal Thermal Cycler, Biorad, SA) containing three technical replicates and three biological replicates.

## Supplementary information

Supplementary Figures and Tables

Data S1

Data S2

Data S3

Data S4

Data S5

Data S6

Data S7

## Data Availability

Data generated or analysed during this study are included in this published article and its supplementary information files. PacBio SMRT reads and Illumina SGS reads generated in this study have been submitted to the BioProject database of National Center for Biotechnology Information (accession number PRJNA503298, http://www.ncbi.nlm.nih.gov). We have requested that our data are held confidential until September 22, 2019. They will not be released to the public until this date, or until the data or accession numbers appear in print, whichever is first.
