# Supplementary material for: SMRT sequencing of full-length transcriptome of seagrasses Zostera japonica
Source: Sci Rep. 2019 Oct 10;9:14537. doi: 10.1038/s41598-019-51176-y (PMC6787188; doi:10.1038/s41598-019-51176-y)
Supplement: Supplementary file 1 — Supplementary Figures and Tables [file 41598_2019_51176_MOESM1_ESM.pdf]

SMRT sequencing of full-length transcriptome of seagrasses *Zostera japonica*

Siting Chen[1*], Guanglong Qiu[1], Mingliu Yang[1]

[1]Guangxi Key Lab of Mangrove Conservation and Utilization, Guangxi Mangrove Research Center, Guangxi Academy of Sciences, Beihai, Guangxi, 536007, China

[*]Corresponding author at: Guangxi Key Lab of Mangrove Conservation and Utilization, Guangxi Mangrove Research Center, Guangxi Academy of Sciences, Beihai, Guangxi, 536000, China

E-mail address: c105043041@126.com

Guanglong Qiu: qalong@163.com

Mingliu Yang: yangmingliu2010@163.com

Legends for supplementary figures and tables

Figure S1. The monitoring data of the bay water temperature.

Figure S2. Schematic of the computational and bioinformatics analysis.

Figure S3. Number and length distributions of 29,058 non-redundant transcripts in *Z. japonica*.

Figure S4. Number and length distributions of predicted ORFs in 29,058 non-redundant transcripts in *Z. japonica*.

Figure S5. Samples sequenced using TGS and SGS.

Table S1. Reads obtained per sample.

Table S2. Orthologous genes in *Arabidopsis* for genes in Figure 7.

Table S3. Maping the entire RNA-seq reads coming from the HiSeq to the *Zostera muelleri* and *Zostera marina* genome.

Table S4. The detection indexes of RNA.

Table S5. List of transcripts used for transcriptome validation via qRT-PCR.

Data S1. PacBio Iso-Seq output analysis.

Data S2. Alternative splicing events.

Data S3. SSR of *Z. japonica*.

Data S4. Long noncoding RNAs.

Data S5. Differently expressed transcripts.

Data S6. Ortholog in *Zostera muelleri*.

Data S7. Ortholog in *Zostera marina*.

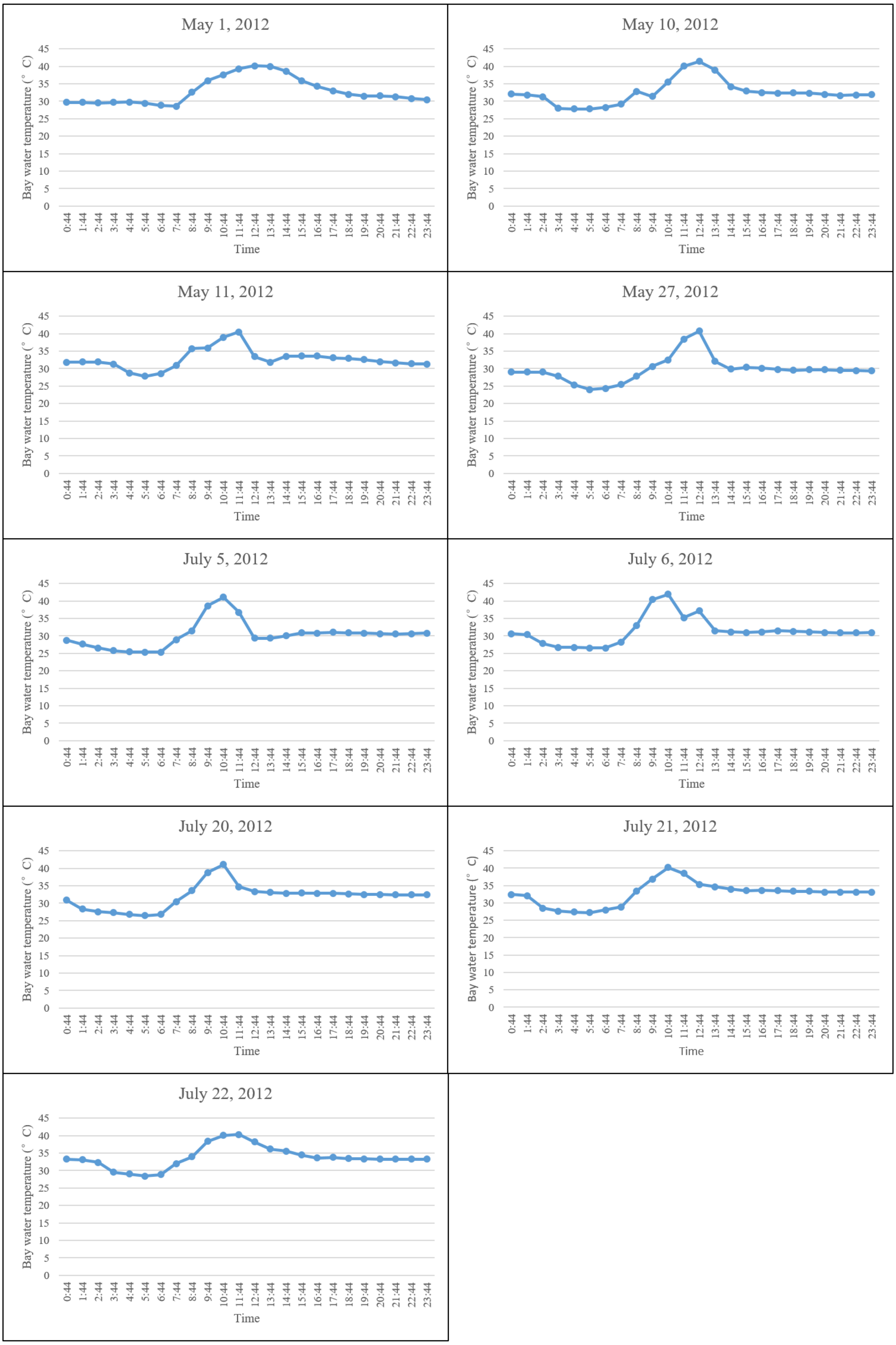

Figure S1. The monitoring data of the bay water temperature.

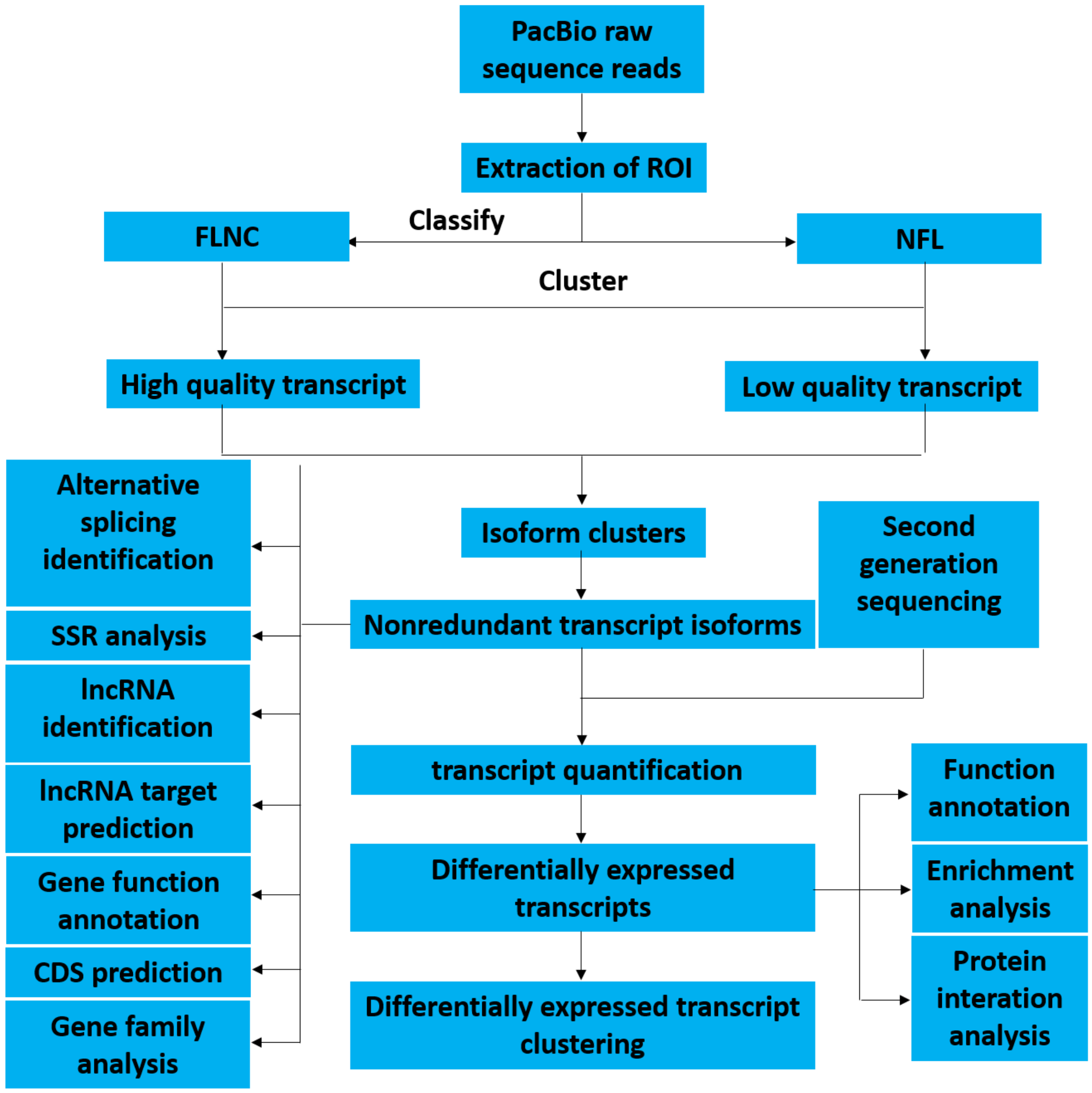

Figure S2. Schematic of the computational and bioinformatics analysis.

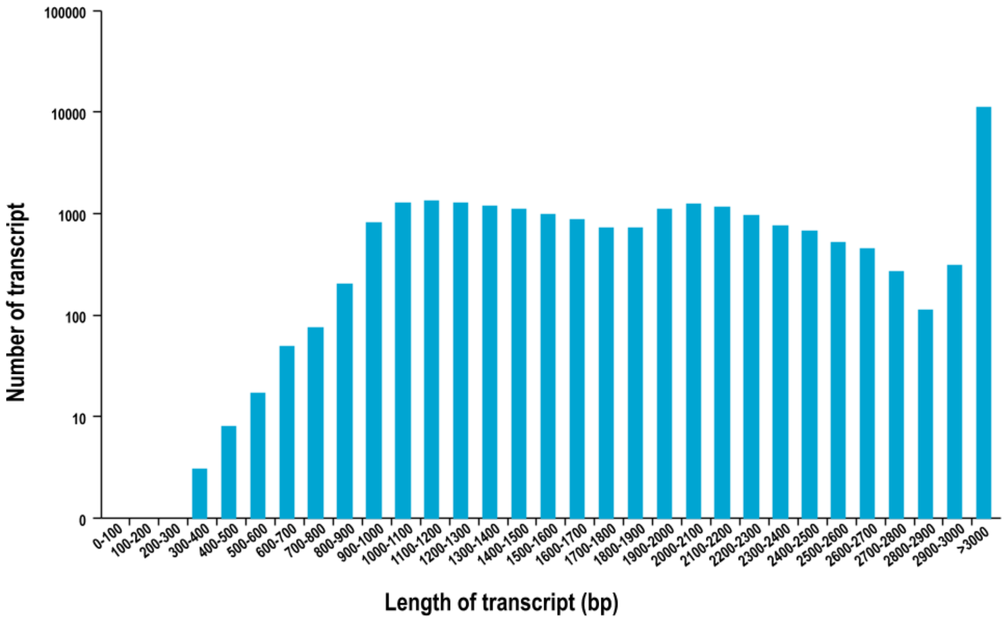

Figure S3. Number and length distributions of 29,058 non-redundant transcripts in *Z. japonica*.

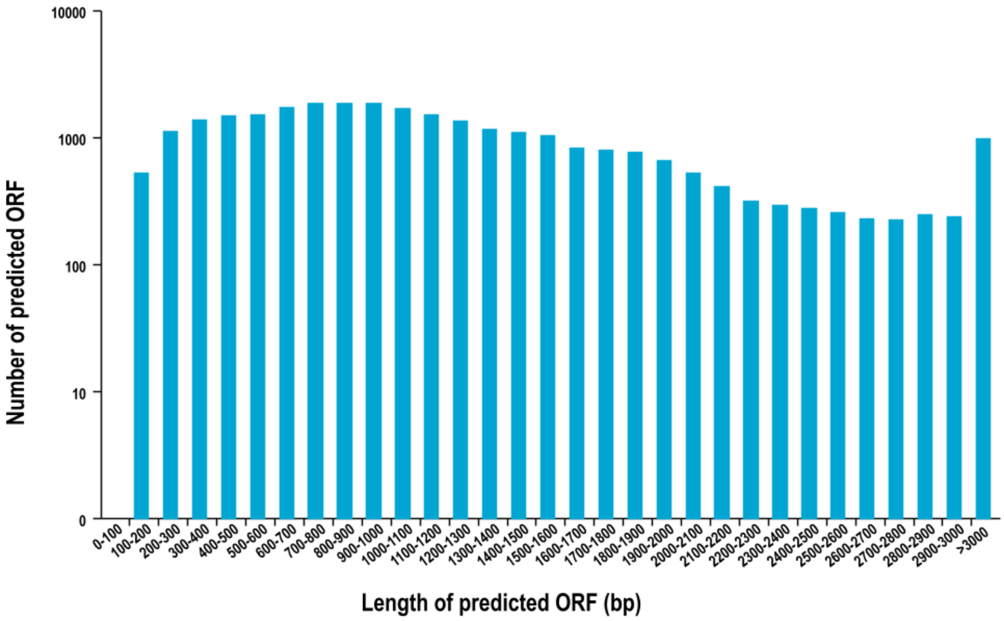

Figure S4. Number and length distributions of predicted open reading frames (ORFs) in 29,058 non-redundant transcripts in *Z. japonica*.

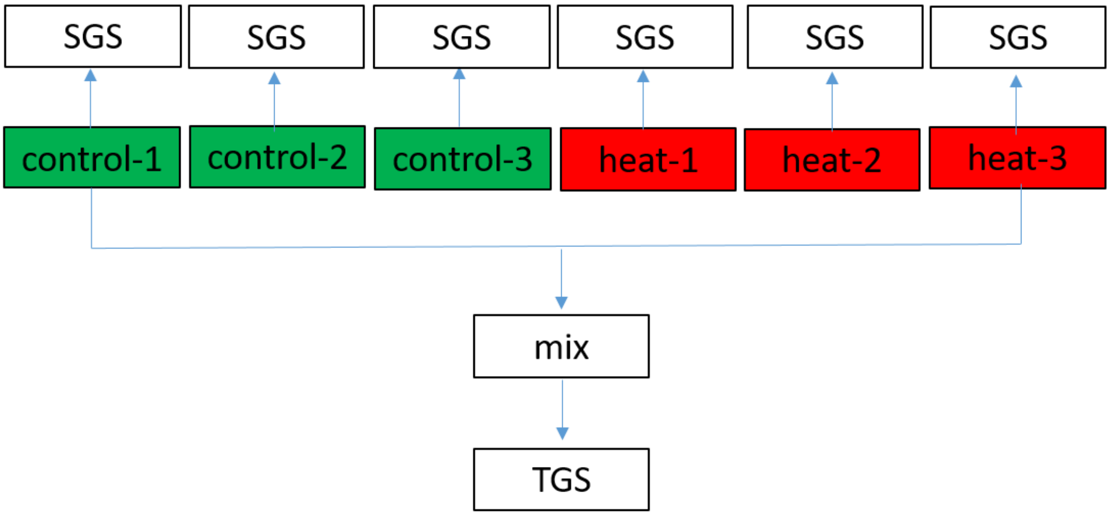

Figure S5. Samples sequenced using TGS and SGS.

Supplementary tables

Table S1. Reads obtained per sample.

Sample name	Reads	Bases	GC(%)	Q30(%)
control-1	21,456,321	6,405,482,938	47.54	94.34
control-2	22,058,509	6,591,953,910	47.01	94.43
control-3	22,505,954	6,718,383,544	47.86	94.40
heat-1	23,147,679	6,916,228,442	48.10	94.43
heat-2	27,634,661	8,248,438,992	48.15	94.28
heat-3	20,654,538	6,159,778,704	47.34	94.23

Table S2. Orthologous genes in *Arabidopsis* for genes in Figure 7.

Differentially expressed genes in *Z. japonica*	Orthologous genes in *Arabidopsis*	Description	Other names
T01.PB1016	AT3G50770	calmodulin-like 41	CML41
T01.PB10694	AT5G02500	encodes a member of heat shock protein 70 family	HSP70-1
T01.PB11340	AT3G07365	Natural antisense transcript overlaps with AT3G46230	
T01.PB11845	AT5G02500	encodes a member of heat shock protein 70 family	HSP70-1
T01.PB11987	AT5G02500	encodes a member of heat shock protein 70 family	HSP70-1
T01.PB12109	AT5G56010	A member of heat shock protein 90 (HSP90) gene family.	ATHSP90-3
T01.PB12151	AT1G79920	Heat shock protein 70 (Hsp 70) family protein	HSP70-15
T01.PB12257	AT3G12580	heat shock protein 70	HSP70
T01.PB12503	AT5G02500	encodes a member of heat shock protein 70 family	HSP70-1
T01.PB12630	AT5G02500	encodes a member of heat shock protein 70 family	HSP70-1

Table S3. Maping the entire RNA-seq reads coming from the HiSeq to the *Zostera muelleri* and *Zostera marina* genome.

Sample	*Zostera muelleri*	*Zostera marina*
control-1	60.43%	4.33%
control-2	60.00%	4.16%
control-3	59.82%	4.38%
heat-1	59.79%	4.71%
heat-2	59.57%	4.17%
heat-3	57.67%	3.59%

Table S4. The detection indexes of RNA.

Sample name	RIN value	28S/18S	baseline	OD260/280	OD260/230
control-1	8.5	1.97	normal	2.17	2.46
control-2	8.9	2.08	normal	2.14	1.86
control-3	7.9	1.31	normal	2.13	1.33
heat-1	8.8	1.95	normal	2.14	1.76
heat-2	8.2	2.03	normal	2.13	1.22
heat-3	8.4	2.09	normal	2.11	1.87

Table S5. List of transcripts used for transcriptome validation via qRT-PCR.

Transcript name	Primer forward sequence	Primer reverse sequence
T01.PB1016	GACGGTCTTCTTGGGTTCG	GACTATGAGCGGTGATTTGG
T01.PB10694	ACTCTTGCGTCGGTGTCTG	TCTCCGTGTCCGTAAATGC
T01.PB11340	GGTACGGGCTCACTTCTAT	TCAGTATGTTCCCATCTTCG
T01.PB11845	CTAAGCGTCTCATCGGTCGTA	TCCGCAGTCTCACGCATCT
T01.PB11987	GAGTTTGGCAACATGACCG	ATGGCAACCTGATTCTTCG
T01.PB12109	CCTAACACCTTCGGCAATA	GTCCACCTCCTCCATCTTG
T01.PB12151	TGGGATTTGATATTGGGAATG	CGCTTGATTTGGGAGATTG
T01.PB12257	TGACCAAGATGCGTGAGAT	GTCAAGACCGTAGGCGATA
T01.PB12503	TAAGAACGCTTTGGAGAACTAC	TTGCTGTCTAGCCAACTGAT
T01.PB12630	GAGCGGTGAGGGCAATGAG	CTTGGTGGGAATGGTGGTG
actin	CACATTTACCACCACAGCC	AACCTCGGGACATCTGAAA
